# Clinical outcomes with use of radiation therapy and risk of transformation in early-stage follicular lymphoma

**DOI:** 10.1038/s41408-022-00620-w

**Published:** 2022-02-10

**Authors:** Fushen Sha, Michelle Okwali, Anna Alperovich, Philip C. Caron, Lorenzo Falchi, Audrey Hamilton, Paul A. Hamlin, Steven M. Horwitz, Erel Joffe, Niloufer Khan, Anita Kumar, Matthew J. Matasar, Alison J. Moskowitz, Ariela Noy, Colette Owens, Lia M. Palomba, Ildefonso Rodriguez-Rivera, David Straus, Gottfried von Keudell, Andrew D. Zelenetz, Joachim Yahalom, Ahmet Dogan, Heiko Schöder, Venkatraman E. Seshan, Gilles Salles, Anas Younes, Connie L. Batlevi

**Affiliations:** 1 Department of Medicine, Lymphoma Service, Memorial Sloan Kettering Cancer Center, New York, NY USA; 2 Currently employed at NEXT Oncology / Texas Oncology, San Antonio, TX USA; 3 Department of Radiation Oncology, Memorial Sloan Kettering Cancer Center, New York, NY USA; 4 Department of Pathology, Hematopathology Service, Memorial Sloan Kettering Cancer Center, New York, NY USA; 5 Department of Radiology, Nuclear Medicine Service, Memorial Sloan Kettering Cancer Center, New York, NY USA; 6 Department of Epidemiology and Biostatistics, Memorial Sloan Kettering Cancer Center, New York, NY USA; 7 Currently employed at AstraZeneca, Wilmington, DE USA

**Keywords:** B-cell lymphoma, Risk factors

## Abstract

Between 1998 and 2009, a total of 295 patients (median age 58, 53% females) with newly diagnosed early-stage follicular lymphoma (FL) were managed at Memorial Sloan Kettering Cancer Center. Approximately half of patients (137, 46%) underwent initial observation and half (158, 54%) immediate treatment: radiation alone (*n* = 108), systemic treatment alone (*n* = 29), or combined modality treatment (*n* = 21). Median follow-up was 8.4 years (range 0.3–17.2), and 10-year overall survival (OS) was 87.2%. OS was similar between initially-observed and immediately-treated patients (hazard ratio [HR]: 1.25, 95% CI: 0.67–2.36, *p* = 0.49). For patients receiving radiation alone, 5-year OS was 98.0%. Patients selected for systemic therapy alone had high-risk baseline features and had shorter OS than patients treated with radiation alone (HR 3.38, 95% CI 1.29–8.86, *p* = 0.01). Combined modality treatment did not yield superior survival compared with radiation alone (*P* > 0.05) but was associated with better progression-free survival (HR 0.36, 95% CI 0.14–0.90, *p* = 0.03). The rate of transformation increased steadily over time and was 4.2% at 5 years and 10.8% at 10 years. This modern-era analysis rationalized the role of initial observation in patients with early-stage FL although patients receiving radiation therapy also demonstrate excellent outcome.

## Introduction

Follicular lymphoma (FL) is the most common indolent lymphoma in the United States [1]. Initial management of FL depends on the stage at presentation. Approximately one-quarter of cases are early-stage (stage I–II) at diagnosis [1, 2]. Though patients with advanced-stage FL are considered incurable and are generally treated with chemoimmunotherapy when treatment is warranted, patients with stage I-II FL can be managed with a number of different approaches.

Initial observation has been reported to be an acceptable treatment option for patients with early-stage FL. However, this conclusion was largely based on small retrospective experiences before the use of modern imaging modalities [3, 4]. The initial observation strategy has not been well validated in a larger patient population where 2-[^18^F]-Fluoro-2-deoxyglucose (FDG) positron emission tomography (PET) integrated with computed tomography (FDG-PET/CT) is routinely available. For patients receiving treatment after diagnosis, radiation therapy (RT) is an effective treatment option but relapse occurs in approximately 35% of cases [5–7]. Furthermore, disease progression primarily outside of the RT field can occur after RT [7, 8]. Studies investigating the benefit of combined modality treatment (CMT) drew distinct conclusions. The British National Lymphoma Investigation randomized trial found the addition of adjuvant oral chlorambucil to RT conferred no survival advantage, whereas the Trans-Tasman Radiation Oncology Group (TROG) found the addition of systemic chemotherapy regimen to RT was associated with longer response duration [9, 10]. In a recent study with early-stage FL patients staged with PET, systemic treatment without rituximab maintenance failed to improve progression-free survival (PFS) [5]. The evidence guiding clinical management of early-stage FL patients in the era of modern imaging modality has been sparse. After completing therapy, patients with FL undergo regular monitoring to capture disease progression and histological transformation. However, the pattern of histological transformation has not been well illustrated in early-stage FL to inform long-term surveillance strategy.

To address these knowledge gaps, we initiated this single-center, retrospective analysis of patients with early-stage FL, including a subpopulation who were staged with PET and bone marrow examination. We aimed to determine survival patterns and risk of transformation in these patients, as well as comparing the clinical outcomes (a) for initial observation >6 months vs immediate treatment, and (b) for immediate treatment with systemic therapy vs radiation therapy vs combined modality therapy.

## Methods

**Fig. 1:**
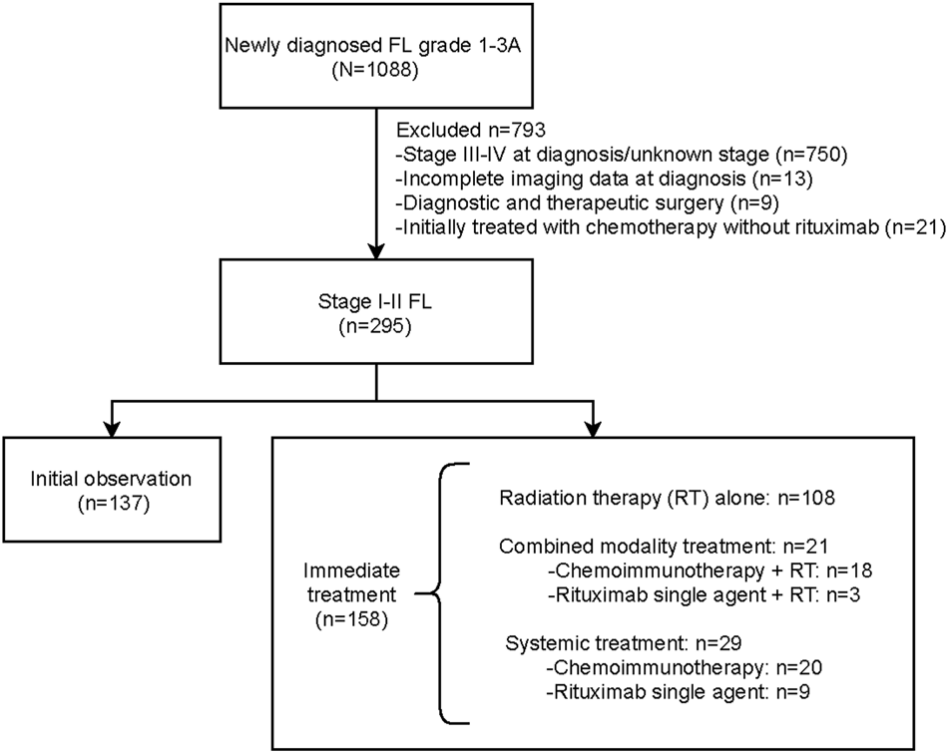
CONSORT diagram of eligible patients with follicular lymphoma. Patients with grade 1–3 A, stage I–II follicular lymphoma (FL) diagnosed between 1998 and 2009 and managed at MSK were identified. FL follicular lymphoma, RT radiation therapy.

The study was approved by the institutional review board of Memorial Sloan Kettering Cancer Center (MSK). We identified 1088 patients with grade 1–3 A FL who were managed at MSK between 1998 and 2009. Study eligibility criteria included age ≥18 years, stage I–II disease, MSK confirmed FL diagnosis, and no prior treatment for FL. We excluded 793 patients from this analysis: 750 had stage III–IV or an unknown stage of disease at diagnosis, 13 had incomplete imaging data at diagnosis, 9 patients had diagnostic and therapeutic surgery, and 21 patients had been treated with chemotherapy without rituximab, which does not represent the current standard of care (Fig. 1). Thus, 295 patients with histologically confirmed FL who were managed and followed at MSK were included in this analysis.

The subpopulation of patients who were completely staged (*n* = 154) had stage I–II disease confirmed with both PET scan and bone marrow examination. The other 141 patients were provisionally staged as stage I–II, i.e., staged with computed tomography without an FDG-PET or a bone marrow biopsy. All pathology was reviewed by MSK pathologists at the time of diagnosis and treatment.

For our analysis, we classified patients based on the length of time between diagnosis and start of treatment. Patients whom we considered to have immediate treatment were started on localized or systemic treatment within 6 months of diagnosis. Conversely, patients initially observed were observed for longer than 6 months.

Overall survival (OS) is measured from the date of diagnosis until death from any causes. PFS is defined as lasting from therapy initiation until disease progression or death from any cause. Multiple imputation was used to impute the Follicular Lymphoma International Prognostic Index (FLIPI) for missing data. We used Cox regression to estimate hazard ratios (HR) and 95% confidence intervals (CI) for associations of treatment modality with OS and PFS, adjusting for imputed FLIPI.

To understand the pattern of disease-specific cause of death and histological transformation of FL following diagnosis, we used a competing risks analysis. For disease-specific cause of death, patients can experience death from lymphoma progression or death from unknown causes. Patient deceased from lymphoma-unrelated causes of death were censored. For histological transformation, a patient can experience one of two mutually exclusive events—pathologically confirmed transformed FL (tFL) or death without tFL. The time origin was set at the time of diagnosis. The cause-specific event rates were plotted against time after diagnosis [11].

Statistical significance was defined as *P* < 0.05, and all statistical analyses were completed with R 3.5.0. Demographics were compared between patients using t-test, Fisher’s exact, and ANOVA test, as appropriate.

## Results

### Patient characteristics

We identified 295 eligible patients diagnosed with grade 1–3 A, stage I–II FL managed at Memorial Sloan Kettering Cancer Center from 1998 to 2009 (Fig. 1). Baseline characteristics at diagnosis are summarized in Table 1. Among 295 patients, 154 patients were staged completely with PET and bone marrow examination. The median follow-up of all 295 eligible patients was 8.4 years (range 0.3–17.2), and median age of all patients was 58 years (interquartile range [IQR] 48–67). More than half of patients were diagnosed with stage I disease (*n* = 172; 58%) and 42% (*n* = 123) had stage II disease. The Follicular Lymphoma International Prognostic Index (FLIPI) risk score at diagnosis was available for 81% of patients (239/295), with low-risk and intermediate/high-risk disease representing 87% and 13%, respectively. Baseline demographics were not significantly different between all eligible patients and patients completely staged with PET and bone marrow examination, except that the completely staged subpopulation was less likely to be initially observed: 36% of completely staged patients *vs*. 46% of all patients (*p* = 0.03) (Table 1, Supplemental Fig. S1).

**Table 1:** Baseline characteristics of patients with grade 1–3 A, stage I–II follicular lymphoma managed at MSK between 1998 and 2009.

	All patients		Completely staged^a^		
	(*N* = 295)		(*N* = 154)		
Characteristic	No.	%	No.	%	*P*
Age: Median (IQR)	58 (48–67)	57 (47–65)	0.25		
Sex					
Female	156	53	78	51	0.69
Male	139	47	76	49	
Stage					
I	172	58	94	61	0.61
II	123	42	60	39	
Grade					
1–2	227	86	121	83	0.39
3 A	36	14	25	17	
Unknown	32	8			
FLIPI					
Low	209	87	117	89	0.74
Intermediate-High	30	13	14	11	
Unknown	56	23			
LDH					
Elevated	25	12	13	11	0.86
Normal	189	88	108	89	
Unknown	81	33			
Hemoglobin					
Decreased	15	6	7	5	1.00
Normal	244	94	130	95	
Unknown	36	17			
Nodal areas					
>4	8	3	3	2	0.76
≤4	287	97	151	98	
Bulky disease (>7 cm)					
Yes	30	16	14	12	0.50
No	160	84	100	88	
Unknown	105	40			
PET staged					
Yes	206	70	154	100	NA
No	89	30	0	0	
SUVmax					
>12	13	10	9	9	1.00
≤12	123	90	90	91	
Unknown	159	55			
Bone marrow negativity					
Yes	220	75	154	100	NA
Unknown	75	25	0	0	
Initial management					
Initial observation	137	46	55	36	0.03
Immediate treatment	158	54	99	64	

^a^Completely staged patients underwent PET scan and bone marrow biopsy at diagnosis.

*IQR* interquartile range, *FLIPI* Follicular Lymphoma International Prognostic Index, *LDH* lactate dehydrogenase, *PET* positron emission tomography, *SUV* standard uptake value.

Baseline features between patients who were initially observed and immediately treated were also compared (Supplemental Table S1). Patients immediately treated were more likely to have stage I disease than initially-observed patients (71% *vs*. 44%, *P* < 0.001), as well as grade 3 A pathology (20% *vs*. 7%, *P* < 0.01) and confirmed bone marrow negativity at diagnosis (84% *vs*. 64%, *P* < 0.001). Other features were not statistically different between the two groups. Interestingly, age at diagnosis was not a major determinant in selecting patients for observation or immediate treatment (Supplemental Fig. S2).

### Baseline demographics by management approaches

Baseline features stratified by the management approaches are shown in Table 2 and Supplemental Table S1. Management approaches included initial observation (*n* = 137) and immediate treatment (*n* = 158). For immediately treated patients, 21 received combined modality treatment (CMT), 108 had radiation therapy alone (RT), and 29 received systemic therapy alone. Sites of radiation therapy are further described in Supplemental Fig. S3. Patients treated with CMT all received radiation therapy, plus either single-agent rituximab (*n* = 3) or rituximab in combination with cyclophosphamide, doxorubicin, vincristine, and prednisone (R-CHOP, *n* = 18). Of the patients who received systemic therapy alone, nine patients received single-agent rituximab, and 20 patients had chemoimmunotherapy. Of the total 158 patients who were immediately treated, only 3% (5/158) patients received rituximab maintenance. Patients treated with RT and CMT had similar baseline characteristics except that more patients treated with CMT had grade 3 A pathology (62% *vs*. 12%, *P* < 0.001, Table 2). Compared with patients treated with radiation therapy alone, patients selected for a systemic regimen alone were more likely to have high-risk baseline features, including stage II disease (86% *vs*. 15%, *p* < 0.001), intermediate-high risk FLIPI score (26% *vs*. 7%, *p* = 0.06), elevated LDH (24% *vs*. 6%, *p* = 0.09), involvement of more than 4 nodal areas (7% *vs*. 0%, *p* = 0.05), bulky disease (defined as lesion > 7 cm, 41% *vs*. 6%, *p* < 0.001), and high SUVmax (defined as SUVmax > 12, 32% *vs*. 2%, *p* = 0.01) (Table 2).

**Table 2:** Baseline characteristics based on management approach.

	Initial observation		Combined modality treatment		RT alone		Systemic treatment		*P*
	(*N* = 137)		(*N* = 21)		(*N* = 108)		(*N* = 29)		
Characteristic	No.	%	No.	%	No.	%	No.	%	
Age: Median (IQR)	58 (49–67)		63 (57–70)		56 (47–66)		60 (53–67)		0.29
Sex									
Female	79	58	13	62	52	48	12	41	0.22
Male	58	42	8	38	56	52	17	59	
Stage									
I	60	44	16	76	92	85	4	14	<0.001
II	77	56	5	24	16	15	25	86	
Grade									
1–2	115	93	8	38	83	88	21	84	<0.001
3 A	8	7	13	62	11	12	4	16	
Unknown	14		0		14		4		
FLIPI score									
Low	94	85	14	88	84	93	17	74	0.06
Intermediate-High	16	15	2	13	6	7	6	26	
Unknown	27		5		18		6		
LDH									
Elevated	13	14	2	13	5	6	5	24	0.09
Normal	83	86	14	88	76	94	16	76	
Unknown	41		5		27		8		
Hemoglobin									
Decreased	10	8	0	0	2	2	3	13	0.07
Normal	112	92	17	100	94	98	21	88	
Unknown	15		4		12		5		
Nodal areas									
>4	6	4	0	0	0	0	2	7	0.05
≤4	131	96	21	100	108	100	27	93	
Bulky disease (>7 cm)									
Yes	13	20	3	19	5	6	9	41	<0.001
No	52	80	13	81	82	94	13	59	
Unknown	72		5		21		7		
PET staged									
Yes	90	66	18	86	76	70	22	76	0.26
No	47	34	3	14	32	30	7	24	
SUVmax									
>12	5	8	1	9	1	2	6	32	0.01
≤12	57	92	10	91	43	98	13	68	
Unknown	75		10		64		10		
Bone marrow negativity									
Yes	87	64	19	90	91	84	23	79	0.001
Unknown	50	36	2	10	17	16	6	21	
Rituximab maintenance									
Yes	—		1	5	0	0	4	14	—
No	—		20	95	108	100	25	86	

*RT* radiation therapy, *OBS* observation, *CMT* combined modality treatment, *IQR* interquartile range, *FLIPI* Follicular Lymphoma International Prognostic Index, *LDH* lactate dehydrogenase, *PET* positron emission tomography, *SUV* standard uptake value.

### Survival after diagnosis

**Fig. 2:**
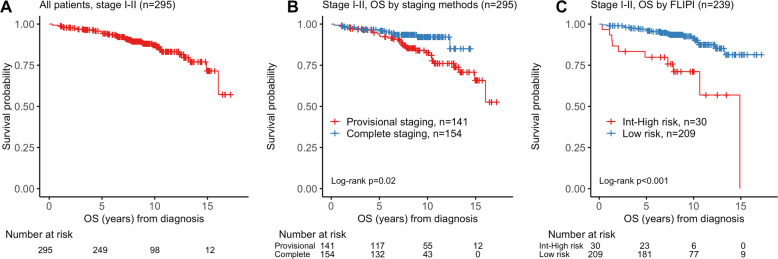
Kaplan–Meier plots of survival after diagnosis. **A** Overall survival (OS) for all patients with stage I-II FL (*n* = 295). **B** OS stratified by staging methods (*n* = 295). **C** OS stratified by the FLIPI risk category at diagnosis (*n* = 239).

The median OS for all comers was not reached based on 39 observed deaths. The 5-, 10-, and 15-year OS rates for all patients were 95.0% (95% confidence interval [CI] 0.925–0.976), 87.2% (95% CI 0.829–0.918), and 71.5% (95% CI 0.595–0.860), respectively (Fig. 2A).

Patients who were completely staged with PET and bone marrow examination had significantly superior OS compared with patients staged provisionally, with median OS being not reached in either group (hazard ratio [HR] 0.45, 95% CI 0.22–0.92, log-rank *p* = 0.02). For completely staged patients, the 5- and 10-year OS rates were 96.7% (95% CI 0.939–0.996) and 92.1% (95% CI 0.873–0.972), respectively. For provisionally staged patients, the 5- and 10-year OS rates were 93.2% (95% CI 0.890–0.976) and 82.6% (95% CI 0.757–0.901), respectively (Fig. 2B).

FLIPI score at diagnosis was prognostic for OS, with patients in the low-risk category demonstrating better survival than patients in the intermediate/high-risk category (HR 0.22, 95% CI 0.10–0.47, log-rank *P* < 0.001). Ten-year OS rates for patients with low and intermediate-high risk FLIPI were 91.4% and 71.1%, respectively (Fig. 2C).

### Impact of initial observation on survival

**Fig. 3:**
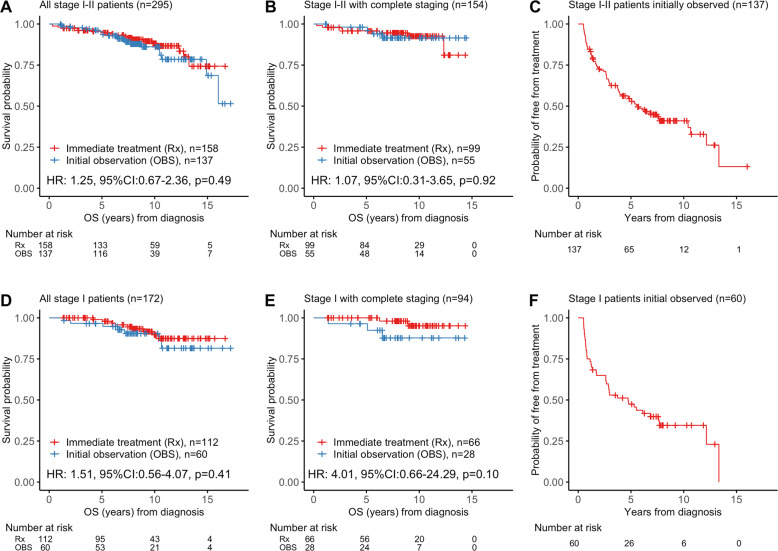
Kaplan–Meier plots of survival and duration of observation after diagnosis. **A** Overall survival (OS) stratified by initial observation versus immediate treatment in all patients with stage I–II FL (*n* = 295). **B** OS stratified by initial observation versus immediate treatment in patients with stage I–II FL who were completely staged with PET scan and bone marrow biopsy at diagnosis (*n* = 154). **C** Duration of observation in all patients with stage I–II FL managed with initial observation (*n* = 137). **D** OS stratified by initial observation versus immediate treatment in all patients with stage I FL (*n* = 172). **E** OS stratified by initial observation versus immediate treatment in patients with stage I FL who were completely staged with PET scan and bone marrow biopsy at diagnosis (*n* = 94). **F** Duration of observation in all patients with stage I FL managed with initial observation (*n* = 60).

Fifty-four percent of all patients (158/295) required immediate treatment after diagnosis, and 46% (137/295) underwent initial observation. Patients completely staged were more likely to be immediately treated after diagnosis, with only 36% of completely staged patients (55/154) managed with initial observation (Table 1). However, the higher rate of initial observation in the provisionally-staged patients did not adversely affect OS for all patients (HR 1.25, 95% CI 0.67–2.36, log-rank *p* = 0.49), nor for the completely-staged subpopulation (HR 1.07, 95% CI 0.31–3.65, log-rank *p* = 0.92) (Fig. 3A, B). The median duration of observation in the initially-observed group (*n* = 137) was 5.62 years (95% CI 3.95–10.41, Fig. 3C).

For all initially-observed stage I patients, OS was similar to that of immediately treated patients (HR 1.51, 95% CI 0.56–4.07, log-rank *p* = 0.41, Fig. 3D). For stage I patients who were completely staged, OS between initially-observed and immediately-treated patients was not significantly different (HR 4.01, 95% CI 0.66–24.29, log-rank *p* = 0.13, Fig. 3E). The median duration of observation for initially-observed stage I patients was 4.73 years (95% CI 2.66-not reached [NR], Fig. 3F). For stage II patients, those who were initially observed also had comparable OS to those who were immediately treated (Supplemental Fig. S4A, B). The median duration of observation for initially-observed stage II patients was 6.90 years (95% CI 4.15-NR, Supplemental Fig. S4C).

For patients who were initially observed and eventually treated (78/137), the median time to first treatment was 2.52 years (range 0.50–13.33 years). For patients who were immediately treated after diagnosis, the median time from diagnosis to treatment was 2.2 months (0.18 years, range 0.00–0.48 years).

### Survival by management approach

**Fig. 4:**
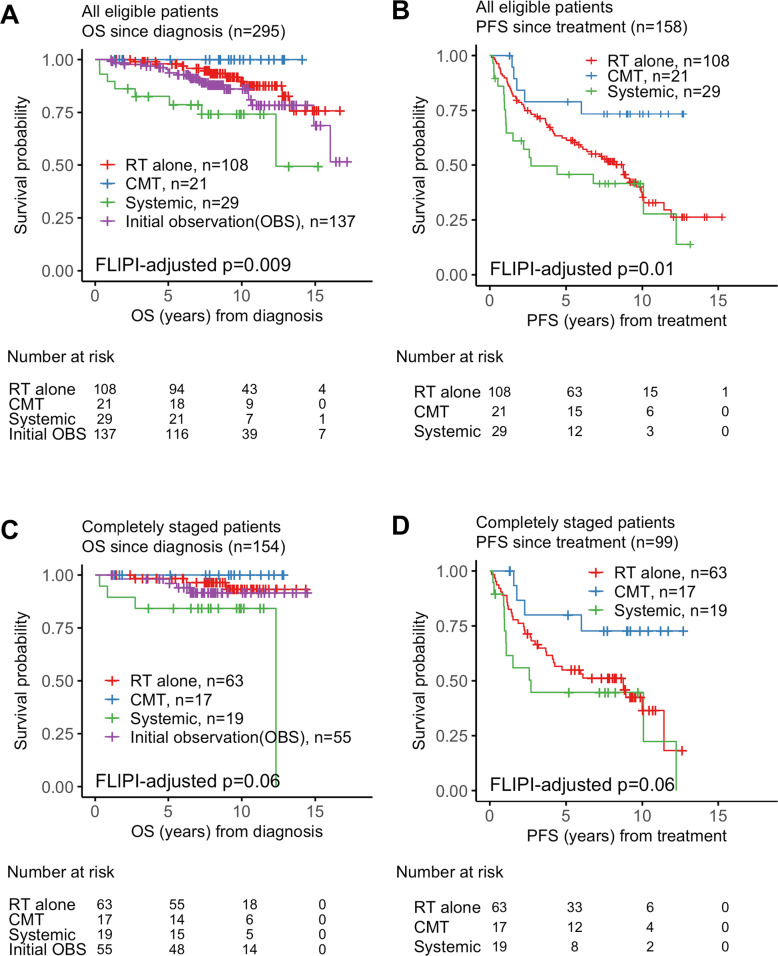
Unadjusted Kaplan–Meier curves with *p*-values adjusted for FLIPI. **A**, **B** Survival analysis to compare outcomes by type of treatment received in all eligible stage I-II patients. **A** overall survival (OS) after diagnosis, **B** progression-free survival (PFS) after treatment, and **C**, **D** Survival analysis to compare outcomes by type of treatment received in stage I-II FL patients who were completely staged. **C** OS after diagnosis. **D** PFS after treatment.

Unadjusted Kaplan–Meier curves illustrate a difference in OS and PFS (Fig. 4). After adjustment for FLIPI, patients treated with CMT had similar OS compared with patients treated with radiation therapy (HR not available due to sparsity of events, *p* = 1.00, Table 3), but had longer PFS (HR: 0.36, 95%: 0.14–0.90, *p* = 0.03, Table 3). Patients selected for systemic treatment had shorter OS vs patients treated with radiation therapy (HR: 3.38, 95%CI: 1.29–8.86, *p* = 0.01, Table 3) despite statistically similar PFS (HR: 1.53, 95%CI: 0.88–2.65, *p* = 0.13, Table 3). Given the prolonged observation time for patients who underwent initial observation in our study, PFS after treatment commencement was not analyzed for patients who were initially observed to avoid lead-time bias and stage migration after observation. Nevertheless, OS since diagnosis after initial observation was not significant abbreviated when compared with patient who were treated with radiation therapy (HR: 1.30, 95%CI: 0.60–2.81, *p* = 0.51, Table 3). Observed trends of survival differences were maintained in subgroup of patients who were completely staged (Table 4).

**Table 3:** Survival analysis in all eligible patients with stage I–II follicular lymphoma based on management approach.

		Initial observation	Combined modality treatment	Radiation therapy alone	Systemic treatment
		(*N* = 137)	(*N* = 21)	(*N* = 108)	(*N* = 29)
OS since diagnosis	5-year (95% CI)	94.6% (0.907–0.986)	100.0% (1.000–1.000)	98.0% (0.953–1.000)	82.6% (0.699–0.977)
	HR^a^ (95% CI)	1.30 (0.60–2.81), *p* = 0.51	NA, *p* = 1.00	Reference	3.38 (1.29–8.86), *p* = 0.01
PFS since treatment	5-year (95% CI)	一	78.9% (0.626–0.996)	62.3% (0.537–0.723)	45.7% (0.304–0.689)
	HR^a^ (95% CI)		0.36 (0.14–0.90), *p* = 0.03	Reference	1.53 (0.88–2.65), *p* = 0.13

^a^Hazard ratio and corresponding *p*-value was adjusted for the Follicular Lymphoma International Prognostic Index (FLIPI). *OS* overall survival, *HR* hazard ratio, *Inf* infinity, *PFS* progression-free survival.

**Table 4:** Survival analysis in patients with stage I–II follicular lymphoma confirmed with positron emission tomography (PET) and bone marrow examination.

		Initial observation	Combined modality treatment	Radiation therapy alone	Systemic treatment
		(*N* = 55)	(*N* = 17)	(*N* = 63)	(*N* = 19)
OS since diagnosis	5-year (95% CI)	98.1% (0.945–1.000)	100.0% (1.000–1.000)	98.4% (0.952–1.000)	84.2% (0.693–1.000)
	HR^a^ (95% CI)	1.70 (0.38–7.71), *p* = 0.49	NA, *p* = 1.00	Reference	4.85 (1.05–22.36), *p* = 0.04
PFS since treatment	5-year (95% CI)	—	80.0% (0.621–1.000)	54.9% (0.438–0.688)	44.7% (0.268–0.748)
	HR^a^ (95% CI)		0.39 (0.14–1.12), *p* = 0.08	Reference	1.50 (0.74–3.06), *p* = 0.26

^a^Hazard ratio and corresponding *p*-value was adjusted for the Follicular Lymphoma International Prognostic Index (FLIPI). *OS* overall survival, *HR* hazard ratio, *Inf* infinity, *PFS* progression-free survival.

Completely staged patients treated with CMT had an excellent 5-year OS rate of 100%. In comparison, the 5-year OS rates after management with either observation, RT, or systemic treatment in completely staged patients were 98.1%, 98.4%, and 84.2%, respectively.

Cumulative incidence curves demonstrating disease-specific cause of death were analyzed by competing risk analysis, where patients experienced either death from lymphoma progression or death from unknown causes. Patients treated with systemic therapy had higher risk of death due to lymphoma progression after adjustment for FLIPI (*p* = 0.001, Supplemental Fig. S5A) whereas the risk of death from unknown causes was similar to the rest of groups after FLIPI adjustment (*p* = 0.73, Supplemental Fig. S5A). The pattern was confirmed in subgroup of patients with complete staging (Supplemental Fig. S5B).

### Transformation

**Fig. 5:**
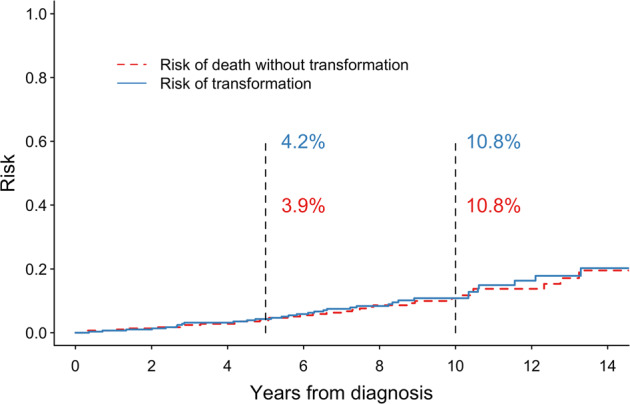
Competing risk analysis of death and transformation. Competing risk analysis to demonstrate the rate of death without histological transformation and the rate of biopsy-proven histological transformation after diagnosis.

We investigated patterns of transformation in early-stage FL. The patterns of biopsy-proven histological transformation and death without transformation are shown in Fig. 5. The rate of transformation, and the rate of death without transformation, steadily increase with increasing time after diagnosis. The risk of transformation at 5 and 10 years after diagnosis was 4.2% and 10.8%, respectively. The risk of death without transformation at 5 and 10 years after diagnosis was 3.9% and 10.8%, respectively. The aforementioned competing risks were not significantly different in patient who were initially observed versus in patient who were immediately treated (Supplemental Fig. S6). Patients selected for systemic treatment alone was observed to have higher risk of transformation at 5 years (18.4%) and 10 years (18.4%, figure not shown).

## Discussion

This study provides important information on early-stage FL and illustrates the survival patterns after management with different approaches, with extended follow-up in the modern era. Patients with deferred treatment underwent prolonged observation with a median duration of 5.62 years, and 43% of patients never required therapy by the time of data collection in 2017. The initial observation did not adversely affect survival compared with the immediately treated population. Similarly, the completeness of staging (presence or absence of PET and bone marrow examination) did not affect the lack of effect of initial observation on OS. After a median follow-up of 8.4 years, the 10-year OS rate was 87% for all eligible patients. In patients with complete staging using PET and bone marrow biopsy, the 10-year OS rate was 92%. Patients treated with RT alone or CMT demonstrated prolonged survival, with 5-year OS rates of 98.0% and 100.0%, respectively. Progression free survival at 5-years for all stage I–II patients was 78.9%, 62.3%, and 45.7% in patients treated with CMT, RT alone, and systemic therapy, respectively but not statistically significant in completely staged patients.

PET has become the new paradigm in FL, and it provides essential information for staging, response assessment, and prognostication [12–16]. PET better identifies both nodal involvement and extra-nodal disease and upstages 24–62% of early-stage FL according to previous studies [13, 14]. Furthermore, PET reveals metabolic heterogeneity, which highlights the niche of suspected transformation to a disease of high aggressiveness. In combination with targeted biopsy, PET potentially influences the choice of initial therapy, the treatment outcome, and exclusion of these patients from accrual into prospective clinical trials or retrospective database analyses. This study confirmed the importance of PET imaging for localized FL. For the 52% of patients who were completely staged with PET and bone marrow biopsy, OS was significantly better than for provisionally staged patients (HR 0.45, 95% CI 0.22–0.92, log-rank *p* = 0.02).

Among patients with advanced-stage FL, treatment in selected patients may be deferred without implication on survival [17]. The role of initial observation in patients with early-stage FL, however, is less evident. In a previous retrospective study of 43 patients with observed stage I-II FL staged in the pre-PET era, the estimated 10-year OS was 85% [4]. The choice of initial observation is desirable when significant co-morbidity is expected from immediate treatment. Our study confirms that initial observation is an appropriate option for early-stage FL patients and is not associated with inferior outcomes.

Major guidelines have recommended RT for early-stage FL, given the radiation-sensitive nature of FL [18–20]. Historically, the 5-year OS rate of early-stage FL was reported to be 82–85% prior to routine radiographic staging with PET. In comparison, the 5-year OS rate for early-stage FL was 93–96% for patients staged in the PET era [5–8]. The survival after RT alone from the current study is again excellent, with 62.3% of patients free from treatment failure 5 years after treatment. Thus, our analysis advocates the incorporation of RT when treatment is indicated. Lymphoma progression generally occurred outside of the RT field, and attempts have been taken to increase long-term disease control for localized FL. Two randomized trials have been conducted to investigate the benefit of adding systemic therapy to RT in early-stage FL. The British National Lymphoma Investigation randomized trial found the addition of adjuvant oral chlorambucil to RT conferred no survival advantage [9]. In a phase III trial developed by the Trans-Tasman Radiation Oncology Group (TROG 99.03), patients with limited-stage FL were randomized to receive either involved-field radiotherapy (IFRT) or IFRT plus six cycles of cyclophosphamide, vincristine, and prednisolone (CVP) with or without rituximab. The addition of systemic chemotherapy was associated with better PFS (HR 0.57; 95% CI 0.34 to 0.95; *p* = 0.033), although this was not reflected in an OS benefit. Enrollment onto TROG 99.03 was over a decade and while the proportion of PET imaging was relatively high, only 50% of patients had PET staging, and rituximab as a component of systemic therapy was only mandatory in a later amendment confounding its modern day applicability [10]. In a recently published analysis from the Australian Lymphoma Alliance, 365 stage I–II FL patients staged with PET demonstrated similar PFS in patients treated with RT alone and systemic therapy without maintenance rituximab (HR 1.32; *p* = 0.96). The addition of maintenance rituximab to radiation therapy improved PFS (HR 0.24; *P* = 0.017) [5]. In the current study, patients treated with CMT demonstrated a similar OS to patients treated with radiation therapy alone. However, CMT was associated with a statistically better PFS in all eligible patients (HR 0.36; *p* = 0.03), although may not be powered to reveal difference in completely staged patients (HR 0.39; *p* = 0.08). In a recent study where early-stage FL patients were evaluated for minimal residual disease (MRD) after involved-field radiotherapy (IFRT), IFRT only induced MRD negativity in 40% of cases, suggesting that radiation therapy alone is insufficient to eradicate FL. Patients with persistent or relapsed MRD received ofatumumab, and future analysis with longer follow-up will better illuminate the role of biomarker-driven approaches in selecting patients who may benefit from combined modality therapy [21].

High-grade transformation to diffuse large B cell lymphoma is an integral part of FL’s natural history and is associated with poor prognosis. Efforts have been made to identify FL patients at high risk for transformation before treatment commencement, including targeted biopsy guided by PET imaging. In our analysis, transformation events were confirmed by centrally reviewed pathological samples rather than solely by clinical criteria, and the competing risk analysis was performed to delineate the transformation patterns in early-stage FL. It revealed that the histological transformation rate steadily increases after diagnosis, with 4.2% of patients experiencing transformation at five years and 10.8% at one decade. Initial observation did not result in a higher rate of transformation compared with immediate treatment. Despite the perception of RT and CMT as possibly definitive therapies for FL, long-term patient surveillance may be required to monitor disease relapse and transformation.

Our study has the inherent weakness of a retrospective analysis. Unlike randomized trials, treatment approach to patients with early-stage FL included in a retrospective database was selected based on various factors presented at diagnosis, including stage, risk category, lesion location, co-morbidities and patients and physicians preferences. The baseline demographics differ across patients managed with different approaches in the current study. Comparing the outcome of patients having received systemic therapy with other groups is hampered by the small size of this group and the presence of patients with high-risk features. Future randomized trials are needed to compare outcomes after different treatment modalities.

In summary, early-stage FL has excellent overall survival after extended follow-up in the modern era. Selection for initial observation is independent of patients’ age and does not adversely affect survival. Patients who received systemic regimen alone were identified to have high-risk baseline features, and overall survival after treatment was inferior to RT alone or CMT. These findings must be interpreted with caution, given the retrospective nature of the study and potential unmeasured confounding biases. Nevertheless, our data demonstrate that RT alone and CMT yielded excellent survival, and that CMT was associated with a better PFS. The definitive conclusion regarding the role of systemic regimen, especially the role of rituximab maintenance, needs to be addressed in future randomized trials incorporating modern imaging modality. Finally, the risk of transformation and disease relapse with early-stage FL increases over time; therefore, continued surveillance of early-stage FL patients is recommended.

## Supplementary information

Supplemental Data
