# Supplementary material for: Clinical outcomes with use of radiation therapy and risk of transformation in early-stage follicular lymphoma
Source: Blood Cancer J. 2022 Feb 10;12(2):29. doi: 10.1038/s41408-022-00620-w (PMC8831497; doi:10.1038/s41408-022-00620-w)

**Clinical Outcomes with Use of Radiation Therapy and Risk of Transformation in Early-Stage Follicular Lymphoma**

Fushen Sha^1^, Michelle Okwali^1^, Anna Alperovich^1^, Philip C. Caron^1^, Lorenzo Falchi^1^, Audrey Hamilton^1^, Paul A. Hamlin^1^, Steven M. Horwitz^1^, Erel Joffe^1^, Niloufer Khan^1^, Anita Kumar^1^, Matthew J. Matasar^1^, Alison J. Moskowitz^1^, Ariela Noy^1^, Colette Owens^1^, Lia M. Palomba^1^, Ildefonso Rodriguez-Rivera^1,6^, David Straus^1^, Gottfried von Keudell^1^, Andrew D. Zelenetz^1^, Joachim Yahalom^2^, Ahmet Dogan^3^, Heiko Schoder^4^, Venkatraman E. Seshan^5^, Gilles Salles^1^, Anas Younes^1,7^, and Connie L. Batlevi^1^

1 Department of Medicine, Lymphoma Service, Memorial Sloan Kettering Cancer Center, New York, NY

2 Department of Radiation Oncology, Memorial Sloan Kettering Cancer Center, New York, NY 1

3 Department of Pathology, Hematopathology Service, Memorial Sloan Kettering Cancer Center, New York, NY

4 Department of Radiology, Nuclear Medicine Service, Memorial Sloan Kettering Cancer Center, New York, NY

5 Department of Epidemiology and Biostatistics, Memorial Sloan Kettering Cancer Center, New York, NY

6 Currently employed at NEXT Oncology / Texas Oncology, San Antonio, TX

7 Currently employed at AstraZeneca, Wilmington, DE

**Corresponding Author:**

Connie Lee Batlevi, MD, PhD

Memorial Sloan Kettering Cancer Center

530 East 74^th^ Street

New York, NY 10021

Phone: 646-608-3707

Email: leec@mskcc.org

**Supplemental Table S1**. Baseline characteristics for stage I-II follicular lymphoma patients based on initial observation versus immediate treatment.

|  | Initial observation | | Immediate treatment | |  |
| --- | --- | --- | --- | --- | --- |
|  | (N=137) | | (N=158) | |  |
| Characteristic | No. | % | No. | % | P |
| Age: Median (IQR) | 58 (49-67) | | 58 (48-67) | | 0.83 |
| Sex |  |  |  |  |  |
| Female | 79 | 58% | 77 | 49% | 0.13 |
| Male | 58 | 42% | 81 | 51% |  |
| Stage |  |  |  |  |  |
| I | 60 | 44% | 112 | 71% | <0.001 |
| II | 77 | 56% | 46 | 29% |  |
| Grade |  |  |  |  |  |
| 1-2 | 115 | 93% | 112 | 80% | <0.01 |
| 3A | 8 | 7% | 28 | 20% |  |
| Unknown | 14 | | 18 | |  |
| FLIPI score |  |  |  |  |  |
| Low | 94 | 85% | 115 | 89% | 0.44 |
| Intermediate-High | 16 | 15% | 14 | 11% |  |
| Unknown | 27 | | 29 | |  |
| LDH |  |  |  |  |  |
| Elevated | 13 | 14% | 12 | 10% | 0.52 |
| Normal | 83 | 86% | 106 | 90% |  |
| Unknown | 41 | | 40 | |  |
| Hemoglobin |  |  |  |  |  |
| Decreased | 10 | 8% | 5 | 4% | 0.18 |
| Normal | 112 | 92% | 132 | 96% |  |
| Unknown | 15 | | 21 | |  |
| Nodal areas |  |  |  |  |  |
| >4 | 6 | 4% | 2 | 1% | 0.15 |
| ≤4 | 131 | 96% | 156 | 99% |  |
| Bulky disease (>7 cm) |  |  |  |  |  |
| Yes | 13 | 20% | 17 | 14% | 0.30 |
| No | 52 | 80% | 108 | 86% |  |
| Unknown | 72 | | 33 | |  |
| PET staged |  |  |  |  |  |
| Yes | 90 | 66% | 116 | 73% | 0.16 |
| No | 47 | 34% | 42 | 27% |  |
| SUVmax |  |  |  |  |  |
| >12 | 5 | 8% | 8 | 11% | 0.77 |
| ≤12 | 57 | 92% | 66 | 89% |  |
| Unknown | 75 | | 84 | |  |
| Bone marrow negativity |  |  |  |  |  |
| Yes | 87 | 64% | 133 | 84% | **<0.001** |
| Unknown | 50 | 36% | 25 | 16% |  |
| Rituximab maintenance |  | |  |  |  |
| Yes | — | | 5 | 3% | — |
| No | — | | 153 | 97% |  |

Abbreviations: FLIPI, Follicular Lymphoma International Prognostic Index; IQR, interquartile range; LDH, lactate dehydrogenase; SUV, standard uptake value.

**Supplemental Figure S1.** Among 206 patients with stage I-II disease confirmed by PET/CT, 154 had confirmed negative bone marrow and 52 had unknown bone marrow status. Survival was similar between these two subgroups (P=0.82, Figure A below). Similarly, survival was similar if the analysis was performed in stage I patients only (P=0.28, Figure B below)


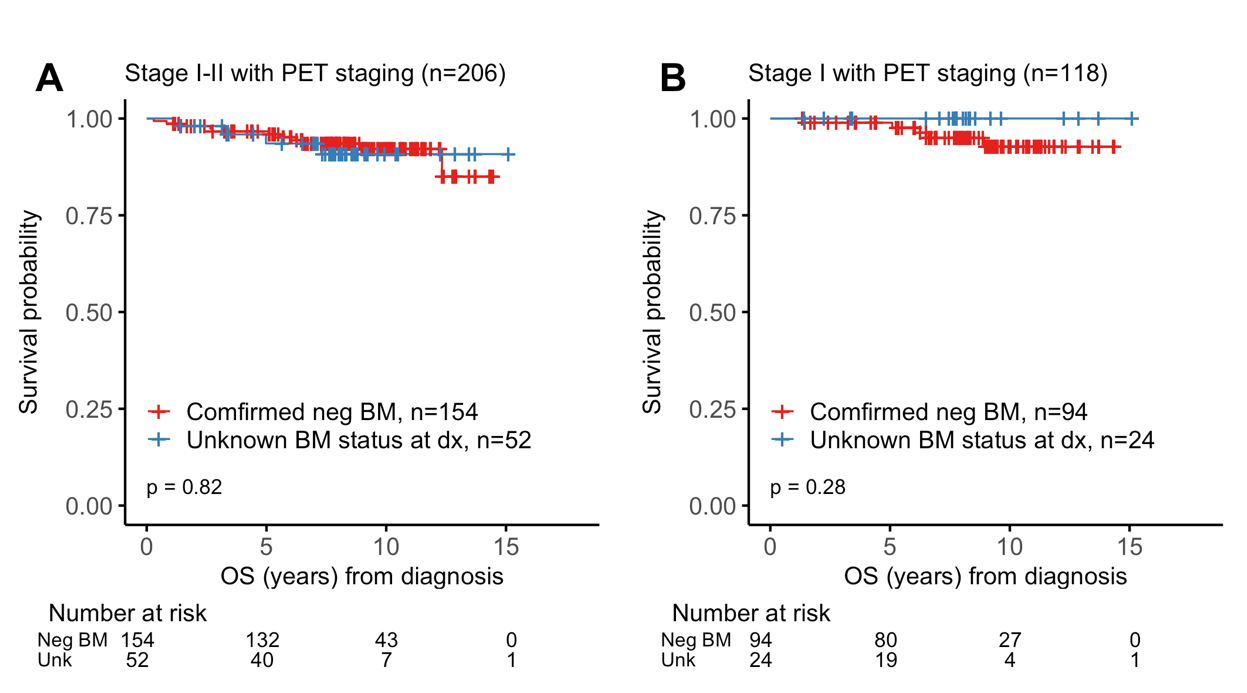


**Supplemental Figure S2**. Selection of patients with stage I (**A**) and stage II FL (**B**) for initial observation by patients’ age at diagnosis.


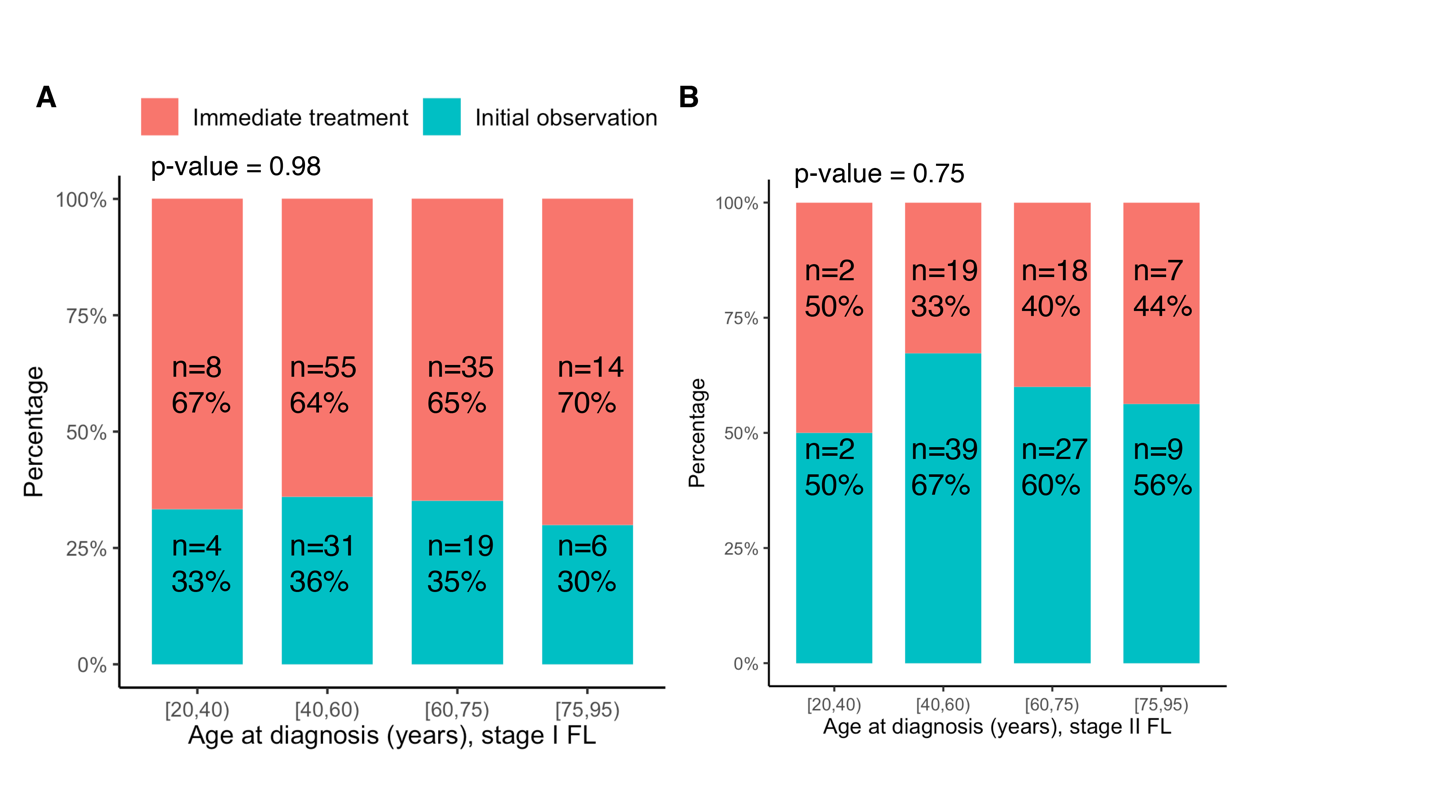


**Supplemental Figure S3.** Sites of radiation therapy. Patients were categorized into three groups based on the site of disease: axial (mesenteric, lung, retroperitoneal, kidney, spleen, pancreas, etc.), peripheral (cervical, extremities, inguinal, axillary, skin, breast, iliac, mandibular, etc.), and unknown site of disease. Patients with axial disease were more likely observed whereas patients with peripheral disease were more likely to receive radiation therapy (**Table below**). No robust conclusion can be drawn given very small patient number, but for axial disease, patients selected for radiation therapy had non-statistically significant superior survival compared with patients selected for observation (**Figure A below**). In peripheral disease, no significant difference in survival was observed following radiation therapy versus observation (**Figure B below**).

| Stage I-II FL patients who were treated with XRT alone or underwent observation (n=245) | | | |
| --- | --- | --- | --- |
|  | Axial (n=48) | Peripheral (n=136) | Unknown (n=61) |
| XRT | 5 (10%) | 101 (74%) | 2 (3%) |
| OBS | 43 (90%) | 35 (26%) | 59 (97%) |


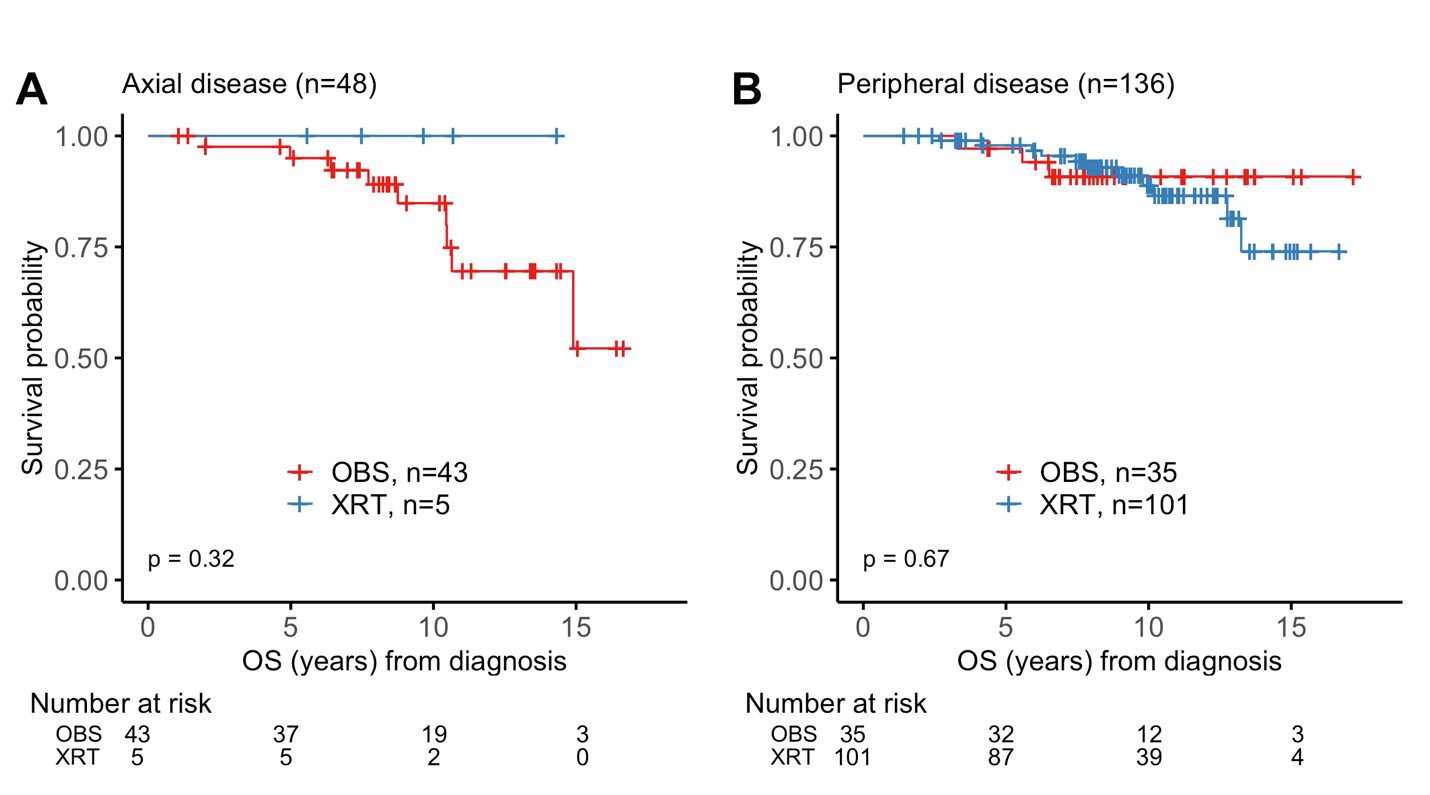


**Supplemental Figure S4.** Kaplan-Meier plots of overall survival (OS) and duration of observation after diagnosis in patients with stage II FL. (**A**) OS stratified by initial observation versus immediate treatment in all patients with stage II FL (n=123). (**B**) OS stratified by initial observation versus immediate treatment in patients with stage II FL who were completely staged with PET scan and bone marrow biopsy at diagnosis (n=60). (**C**) Duration of observation in all patients with stage II FL managed with initial observation (n=77).


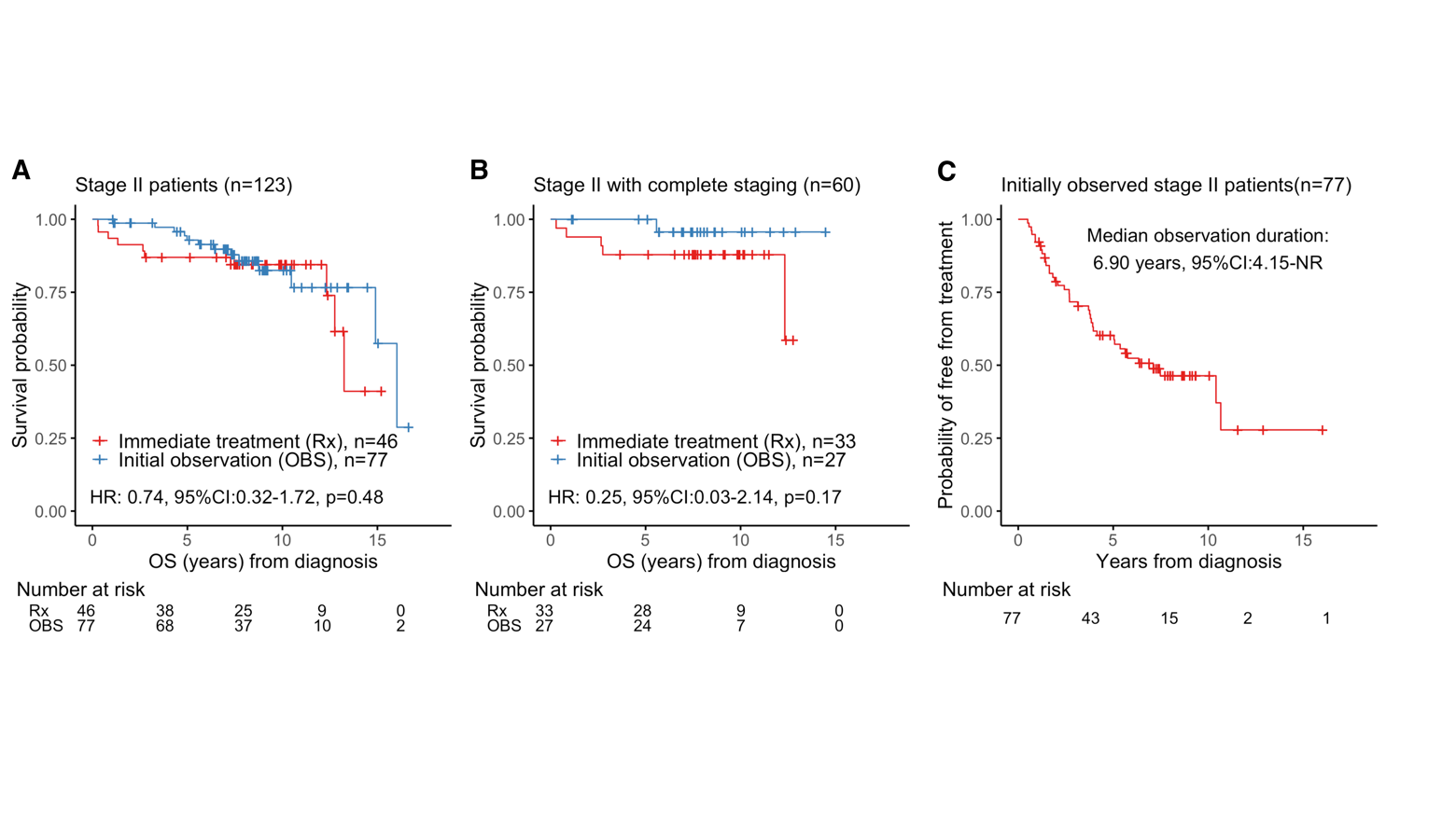


**Supplemental Text.** Progression patterns of relapse was evaluated using time to next treatment. For patients treated with CMT, the median TTNT was not available due to a small number of events (n=4). For patients initially observed, treated with XRT, and treated with systemic therapy, the median TTNT were 7.77 years, 11.62 years, and 7.21 years, respectively. Among 137 patients with stage I-II FL who were initially observed, 78 required treatments later. The distribution and extensiveness of the progression of disease was not completely available for all patients. Nevertheless, among these 78 patients, 55 required systemic therapy (Rituximab in combination with chemotherapy, n=23; Rituximab in combination with chemotherapy and XRT, n=4; Rituximab single agent, n=26; Rituximab single agent in combination with XRT, n=2).

**Supplemental Figure S5.** Competing risk analysis showing disease-specific cause of death

in patients with stage II FL. (**A**) In all patients. (**B**) In patients who were completely staged with PET scan and bone marrow biopsy at diagnosis.


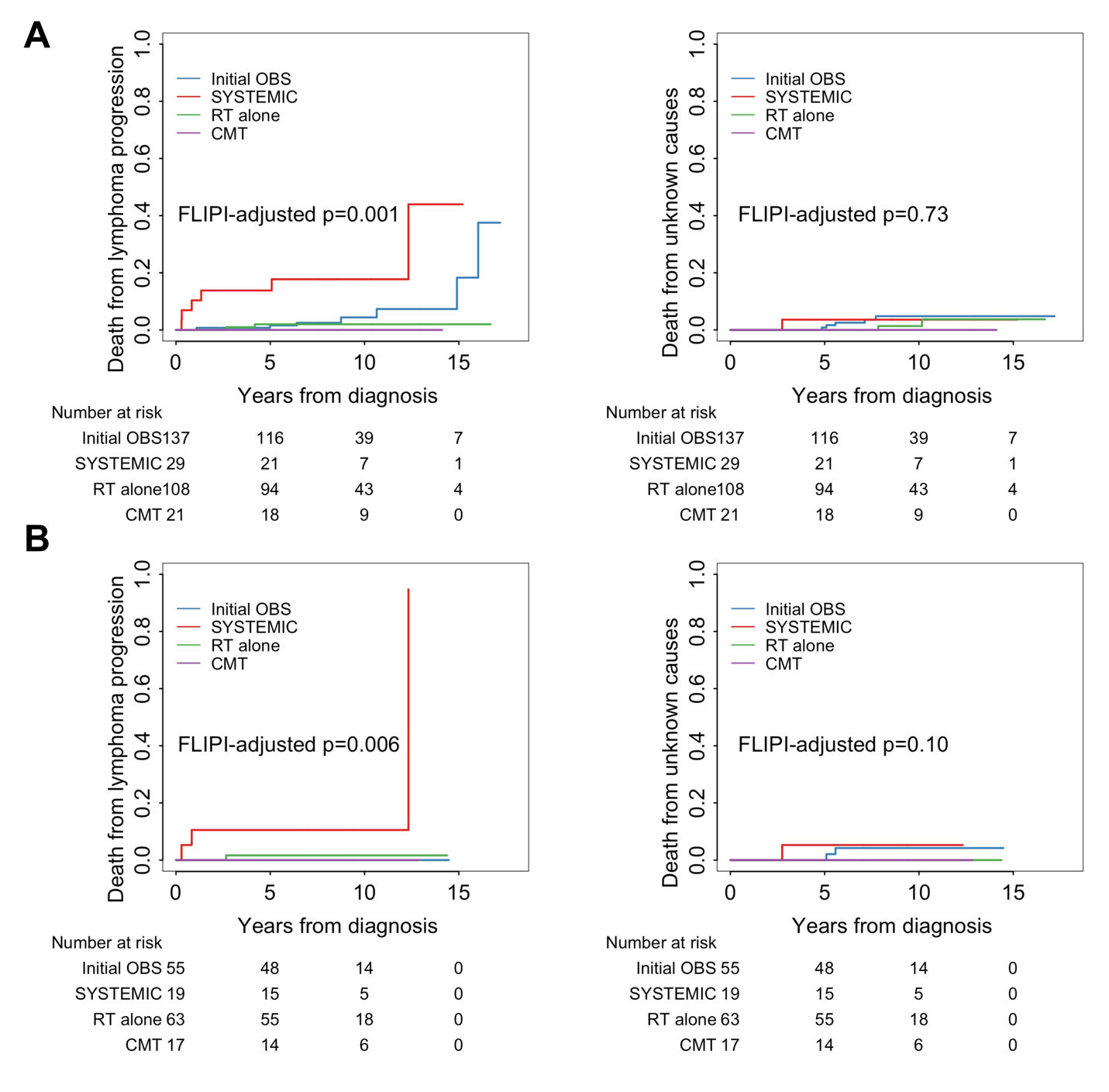


**Supplemental Figure S6.** For patient who were initially observed versus patient who were immediately treated, the rate of death without histological transformation and the rate of biopsy-proven histological transformation after diagnosis were compared using competing risk analysis.


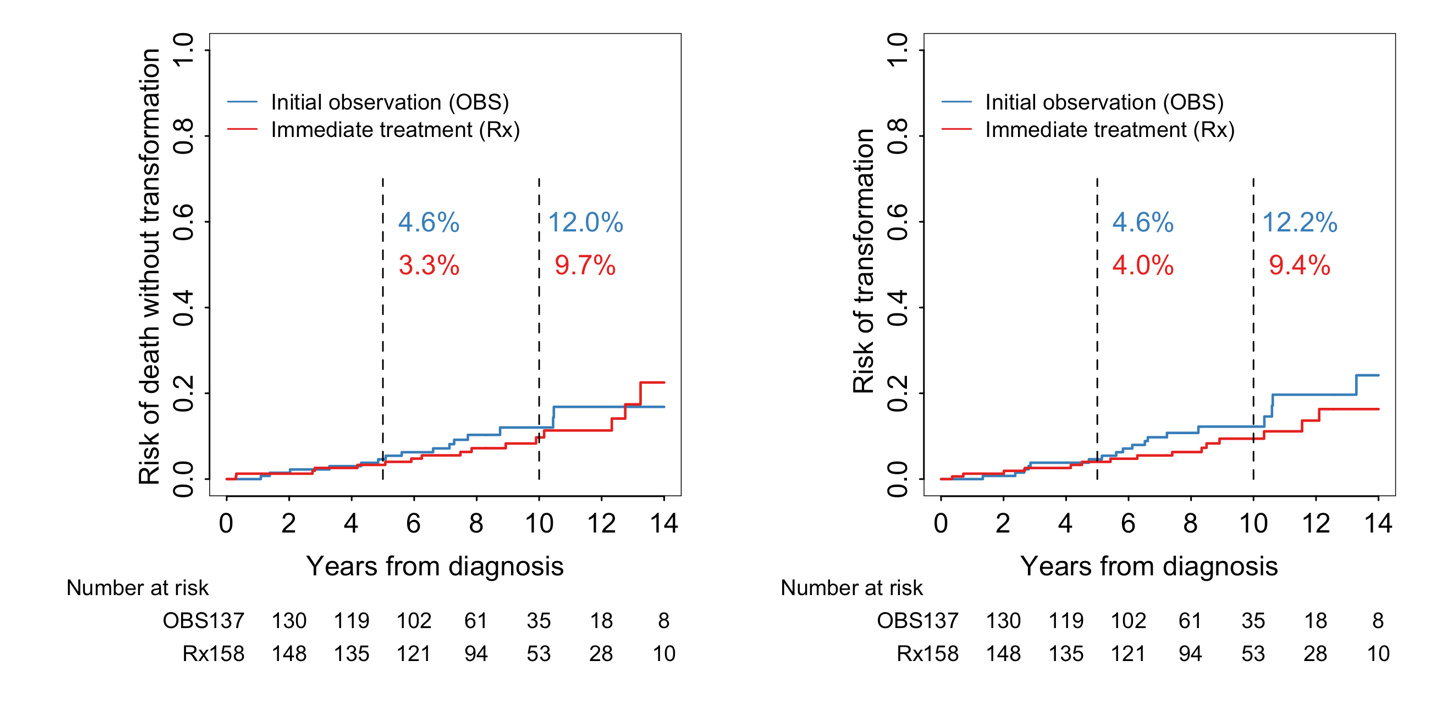

Supplement: Supplementary file 1 — Supplemental Data [file 41408_2022_620_MOESM1_ESM.docx]
